# Plastic Accumulation in the Mediterranean Sea

**DOI:** 10.1371/journal.pone.0121762

**Published:** 2015-04-01

**Authors:** Andrés Cózar, Marina Sanz-Martín, Elisa Martí, J. Ignacio González-Gordillo, Bárbara Ubeda, José Á. Gálvez, Xabier Irigoien, Carlos M. Duarte

**Affiliations:** 1 Departamento de Biología, Facultad de Ciencias del Mar y Ambientales, Universidad de Cádiz, Campus de Excelencia Internacional del Mar, E-11510, Puerto Real, Spain; 2 Department of Global Change Research, IMEDEA (CSIC-UIB), Instituto Mediterráneo de Estudios Avanzados, Miquel Marqués 21, 07190, Esporles, Spain; 3 Departament de Geologia Dinàmica, Geofísica i Paleontologia, Facultat de Geologia, Universitat de Barcelona, 08028, Barcelona, Spain; 4 Red Sea Research Center, King Abdullah University of Science and Technology, Thuwal, 23955–6900, Kingdom of Saudi Arabia; The Evergreen State College, UNITED STATES

## Abstract

Concentrations of floating plastic were measured throughout the Mediterranean Sea to assess whether this basin can be regarded as a great accumulation region of plastic debris. We found that the average density of plastic (1 item per 4 m^2^), as well as its frequency of occurrence (100% of the sites sampled), are comparable to the accumulation zones described for the five subtropical ocean gyres. Plastic debris in the Mediterranean surface waters was dominated by millimeter-sized fragments, but showed a higher proportion of large plastic objects than that present in oceanic gyres, reflecting the closer connection with pollution sources. The accumulation of floating plastic in the Mediterranean Sea (between 1,000 and 3,000 tons) is likely related to the high human pressure together with the hydrodynamics of this semi-enclosed basin, with outflow mainly occurring through a deep water layer. Given the biological richness and concentration of economic activities in the Mediterranean Sea, the affects of plastic pollution on marine and human life are expected to be particularly frequent in this plastic accumulation region.

## Introduction

Recent studies have demonstrated the existence of five large-scale accumulation regions of floating plastic debris in the oceans corresponding to each of the subtropical gyres located at either side of the Equator [1–6]. Ocean currents transport floating plastic released from terrestrial (e.g., coastal cities, rivers, and tourist beaches) and maritime (e.g., vessels, and at-sea platforms) sources to central convergence zones in the open ocean where these materials accumulate [7–9]. This process results in surface concentrations of buoyant plastic up to the order of kilograms (or millions of pieces) per km^2^ in the center of ocean gyres, while open-ocean concentrations outside the gyres only occasionally reach a few grams (or thousands of pieces) per km^2^ [1–5, 10, 11].

However, the assessment of marine plastic pollution is relatively recent, and extensive areas of the ocean remain yet unexplored, including regional semi-enclosed seas located in basins with intense use of plastic. This is the case of the Mediterranean Sea. Its shores house around 10% of the global coastal population (ca. 100 million people within the 10-km coastal strip [12]). The basin constitutes one of world´s busiest shipping routes [13], and receives waters from densely populated river catchments (e.g., Nile, Ebro, and Po). Furthermore, the Mediterranean Sea is only connected to the Atlantic Ocean by the Strait of Gibraltar and has a water residence time as long as a century [14]. Estimating both terrestrial and maritime inputs, Lebreton and coworkers modeled the transport and distribution of floating debris in the ocean [8]. The model simulations identified the Mediterranean Sea as a potentially important accumulation zone at the global scale. Recently, the calibration of this model using a global dataset was applied to estimate the surface plastic load in the Mediterranean Sea at 23,150 tons [6].

The abundance of plastic debris floating in Mediterranean waters was first reported by Morris in 1980 [15]. Using a quantitative visual survey, he reported around 1,300 plastic items per square kilometer in a central region of the basin. However, all other visual counts carried out in different regions of the Mediterranean since then have reported fewer than 200 items per square kilometer [16–19]. Surface pollution has also been quantified using surface net tows, allowing for the detection of smaller plastic sizes, in coastal areas of northwestern Italy, southern France [6, 20], and western Sardinia [21]. These studies reported concentrations ranging from tens of thousands to hundreds of thousands of items per square kilometer, suggesting an abundant presence of buoyant plastic debris in the basin. In the present work, we have carried out extensive sampling across the Mediterranean Sea basin in order to provide a first-order approximation of the magnitude of the plastic pollution in the surface waters of the Mediterranean. Plastic concentrations found in this Sea are compared with those reported for the five regions of plastic debris accumulation in the open ocean.

## Material and Methods

Floating plastic debris was sampled across the Mediterranean basin in May 2013 on board the Spanish R/V *Angeles Alvariño*. Geographical coordinates and dates of sampling are available at http://doi.pangaea.de/10.1594/PANGAEA.842054. Permission for navigation and research operations in exclusive economic zones of the Mediterranean Sea was granted from the Governments of Spain, Greece, France, Italy and Cyprus. Sampling did not involve endangered or protected species.

Plastic debris in surface waters was collected with a neuston net (1.0 × 0.5 m mouth, 0.2 mm mesh) towed at 2–3 knots for periods of ~15 min. A total of 28 sites were sampled using 39 net tows. The material collected in each tow was resuspended in 20 μm-filtered seawater and floating plastic debris was carefully picked from the water surface with the aid of a dissecting stereomicroscope. This examination of the samples was repeated at least twice to ensure the detection of all of the smallest plastic particles. Plastics extracted from the seawater samples were washed with deionized water and dried at room temperature before being weighted. Plastic items were assigned to five product type categories: industrial pellets (the raw form of plastic) and granules (likely derived from cosmetic and cleansing products); thin films (bags, wrappings, or pieces of them); fishing threads (including fishing lines and plastic fibers released from fishing nets); foamed plastic (termed here “foam”); and rigid manufactured items or pieces of them (all termed as “fragments” as the vast majority correspond to pieces from broken objects). Any fibers suspected of being of a textile origin were excluded from the analysis because they could be airborne contamination from clothing during the sampling or processing [22]. Potential textile fibers were identified according to shape and rigidness. They typically ranged from hundreds of microns to centimeters in length and from one to few tens of microns in width, being easily folded; whilst pieces of fishing threads are wider and generally straight in shape (S1 Fig). Tar particles were particularly abundant in relation to open ocean samples collected with the same method in a former study [5], likely due to the high maritime traffic across the Mediterranean. Nevertheless, tar particles were also excluded from the analysis.

The maximum linear length of the plastic items was measured under an optical microscope using the image processing NIS-Elements software, whereas large plastic objects were measured with a ruler. A total of 3,901 plastic items were measured and separated into 28 size classes to build a size distribution. Narrower bins were used to describe the size structure of the smaller plastics. Therefore, size limits of the bins were set following a 0.1-log series of linear length. The width of the uppermost bin extended from 10 cm to 100 cm (the width of the net mouth) due to the relatively low abundance of plastic items in this size interval. To render plastic counts per bin independent of the width of the bin, the abundance of plastic items for each bin was normalized by the bin width. These results were compared with the plastic size distribution found in our previous study of the open ocean [5].

Plastic concentrations per surface area were calculated by dividing the total number and dry weight of plastics collected in each tow by the area towed. This area was derived from the volume of filtered seawater during the tow, measured with a flowmeter at the net, and the submerged area of the net mouth (1.00 m x 0.25 m). Given that wind stress can extend the vertical distribution of buoyant plastic debris below the surface sampling layer (0.25 m deep), surface plastic concentrations derived from tows carried out with average friction velocity in water (u*) > 0.6 cm s^-1^ (54% of the tows) were adjusted following the model proposed by Kukulta et al. [23]. The model provides wind-adjusted numerical concentrations from u* and the numerical concentrations measured in the surface tows. Wind-adjusted abundances were converted to mass concentrations using an empirical correlation based on simultaneous measurements of total weight and abundance of plastic in 609 worldwide tows (S2 Fig). Adjusted and non-adjusted concentrations of plastic measured in the Mediterranean Sea are reported at http://doi.pangaea.de/10.1594/PANGAEA.842054.

Finally, to analyze the plastic abundance in the Mediterranean basin, we combined our data with the regional surveys carried out by Collignon and coworkers in coastal areas of Ligurian Sea in July 2010 [20], and by de Lucia and coworkers in the western coast of Sardinia in July 2012 and July 2013 [21]. Samples collected by Collignon et al. [20] at high wind speeds (located on the northwestern Mediterranean coast) were discarded. These additional data (33 net tows) were spatially averaged over grid cells of 1° in both latitude and longitude (11 grid cells) to avoid giving excess weight to sites with higher sampling density. Overall, the data compilation of plastic concentrations included 72 net tows, resulting in a spatial grid composed of 39 grid cells across the Mediterranean.

## Results and Discussion

**Fig 1:**
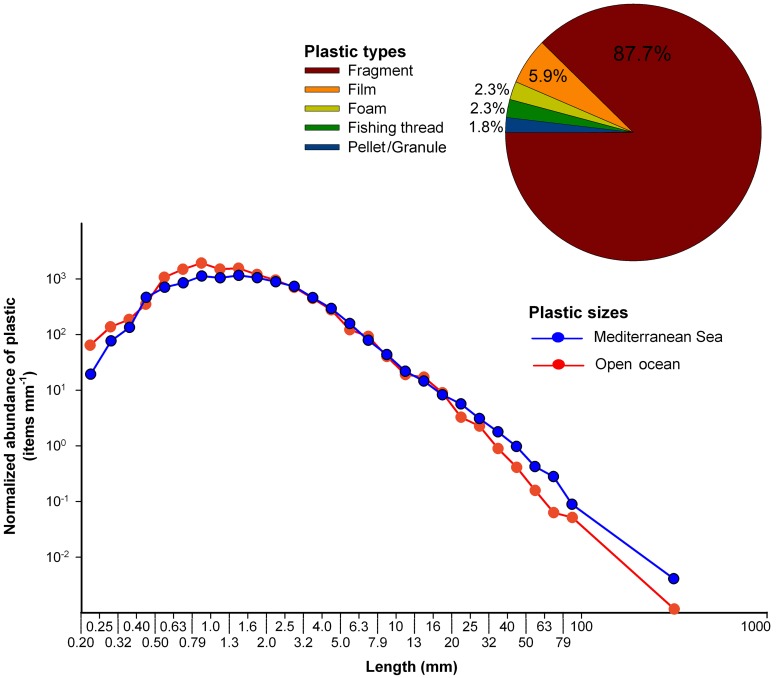
Size distribution and aspect of the floating plastic debris collected in the Mediterranean Sea. The size distribution of plastic debris in the Mediterranean (*n* = 3,901 plastic items; this study) is compared to those measured for plastic accumulation regions in the open ocean (*n* = 4,184 plastic items; [5]). Both plastic size distributions were obtained using the same methodology. Horizontal axis shows the size limits of the bins in logarithmic scale. Because the width of the bins is not uniform, the vertical axis (i.e., normalized abundance of plastic) is shown as number of plastic items divided by the width of the bin (in millimeters). In the comparison of the plastic size distributions in Mediterranean and open-ocean waters, note the logarithmic scale of the vertical axis. The percentages (in abundance) of plastic types (pellets/granules, films, fishing threads, foam, fragments) found in the Mediterranean Sea are shown in the chart at the top right corner.

We found plastic debris in all surface net tows carried out in the Mediterranean Sea. Five different types of plastic items were identified (pellets/granules, films, fishing threads, foam, fragments), with the majority of items being fragments of larger rigid objects (87.7%, e.g. bottles, caps) and thin films (5.9%; e.g. pieces of bags or wrappings) (Fig. 1). Eighty-three percent of the total number of items collected in our nets was smaller than 5 mm in length, commonly referred to as microplastics. The shape of the plastic size distribution was similar to those found in the accumulation zones in the open ocean, with a gradual increase in plastic abundance toward small sizes and a gap below 1 mm (Fig. 1), suggesting removal of plastic items below 1 mm in size from the surface as suggested for the open ocean [5]. However, some differences become apparent in the lowest and highest parts of the size distribution. The proportion of plastic below 2 mm was lower in the Mediterranean Sea than in the open ocean, while the relative abundance of plastic in all size bins above 20 mm was higher in the Mediterranean.

**Fig 2:**
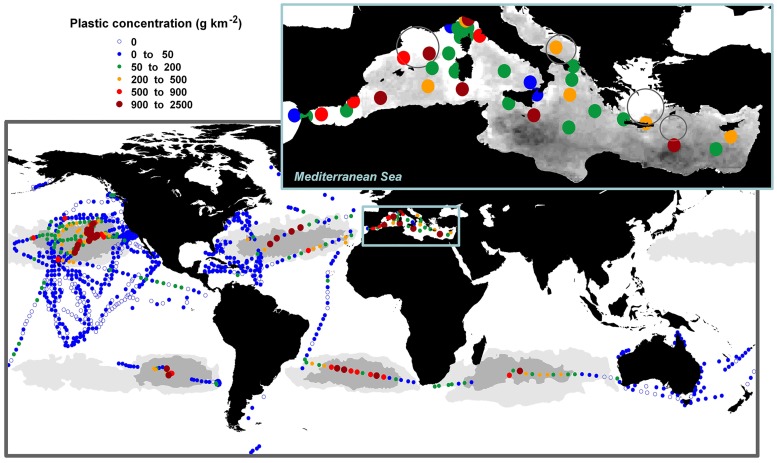
Concentrations of plastic debris in surface waters of the Mediterranean Sea at basin scale (zoomed in the top right corner inset), and compared to the plastic concentrations reported for the global ocean. Gray-scale base map in the Mediterranean basin shows the relative surface plastic concentrations predicted by numerical modeling [8]. Darker areas are predicted to have higher concentrations. Major areas of deep-water formation in the Mediterranean are also shown with black circles [24]. In the global map, dark and light gray areas represent inner and outer accumulation zones, respectively, modeled for the five subtropical gyres; white oceanic areas represent non-accumulation zones [7]. Mediterranean map compiles data from the present study and from ref. [20, 21], while open-ocean map compiles measurements of plastic concentrations from ref. [1–5, 10, 11]. Outside the inner accumulation zones, the open-ocean also includes concentrations reported without correction by wind effect (see details in ref. [5]).

At the basin scale, the spatial distribution of plastic concentrations was irregular, with the highest concentrations scattered throughout the basin. There was no clear association with the main areas of deep-water formation [24] or with the accumulation areas predicted from particle tracking models [8], although high plastic concentrations were generally measured in both of these areas (Fig. 2). The patchy plastic distribution suggests that the variability in the Mediterranean surface circulation [24, 25] hampers the formation of stable plastic retention areas into the basin. A fraction of the variance in the measurements could be related to short-scale sources of variability. The multiple point sources of pollution in the Mediterranean basin could significantly control local plastic concentrations in the short term. High plastic concentrations were found in local shelf areas near population centers (i.e., Portofino, Italy [20]), although a significant correlation between plastic concentration and distance from coast was not found for the whole dataset (slope = +1.04 ±0.91 g km^-1^, *R* = 0.1843, *P* = 0.2617). The small-scale spatio-temporal variability (at days and tens of km) associated with wave- and wind-driven turbulence must also affect the variability in the measurements and account for deviations from model predictions [8, 26]. As a result, the spatio-temporal resolution of the dataset used here precludes as yet a robust analysis of the linkage between hydrodynamics or pollution sources with the observed plastic distribution into the basin.

**Fig 3:**
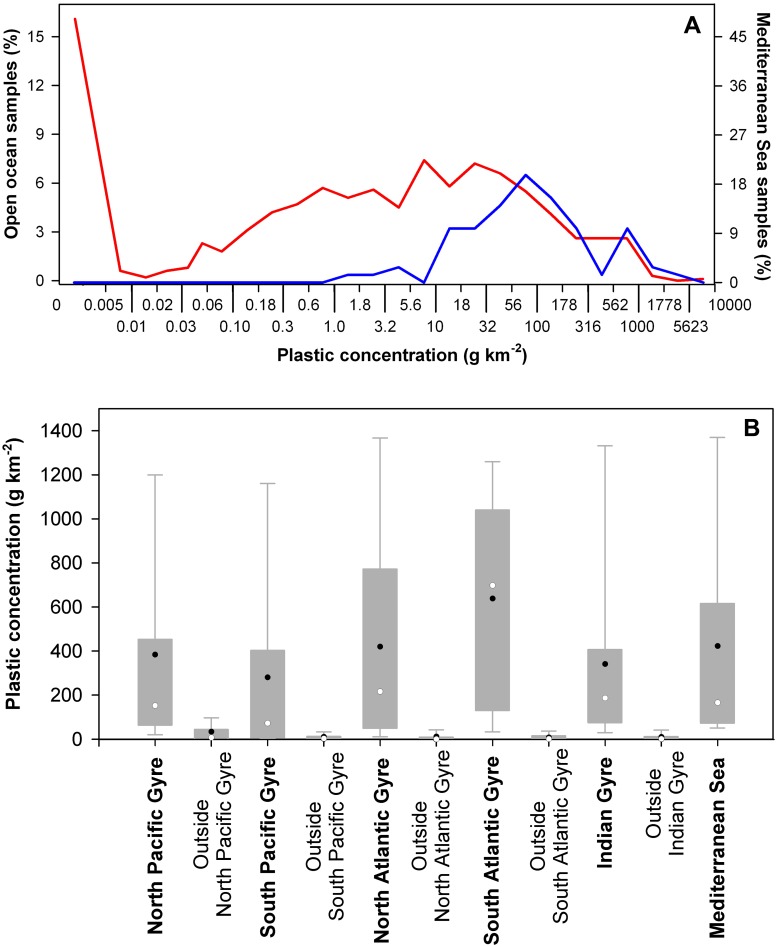
Ranges of surface plastic concentrations measured in the Mediterranean Sea, and reported for the open ocean. A) Frequency distribution of the measurements of plastic concentrations in the Mediterranean Sea (*n* = 72) and in the global ocean (*n* = 1760). Mediterranean measurements (blue line) are from the present study and ref. [20, 21]; ocean measurements (red line), including the five plastic accumulations in the subtropical gyres, were compiled from ref. [1–5, 10, 11]. All these data are mapped in Fig. 2. Size limits of the bins, shown in the horizontal axis, followed a 2.5-log series of plastic concentration (in g km^2^). B) Surface plastic concentrations measured in the Mediterranean Sea, and reported for the inner accumulation zone of the five subtropical gyres (dark gray areas in Fig. 2) [1–5]. Ranges of concentrations outside the convergence zone of each subtropical gyre (white areas in Fig. 2) are also shown for comparative purposes. The boundaries of the boxes indicate the 25th and 75th percentiles, the whiskers above and below the boxes indicate the 95th and 5th percentiles, and the black and white dots mark mean and median respectively. All data in this analysis include correction by wind effect.

Regardless, consideration of the Mediterranean Sea in the context of the global-scale distribution of plastic pollution clearly identifies it as a region of particularly high plastic concentration (Fig. 3). Net sampling sites across the Mediterranean basin showed plastic concentrations ranging from 22 to 1934 g km^-2^, with most of the sites (92%) presenting high concentrations (> 50 g km^-2^) in relation to the range measured for the global ocean (Fig. 2). The average plastic concentration in Mediterranean surface waters was 423 g km^-2^ (243,853 items km^-2^, as numerical concentration), comparable to the average concentrations measured in the inner accumulation zones of the subtropical ocean gyres, which ranged from 281 to 639 g km^-2^. The Mediterranean Sea covers 2.5 millions of km^2^, within the spatial range of the inner accumulation zones of the ocean gyres, ranging from 1 to 5 millions of km^2^ in the global circulation models [7–9]. Hence, the total load of floating plastic debris in the Mediterranean is comparable to that in the accumulation zone of the five subtropical gyres, and this Sea can be considered as an additional great accumulation zone of floating plastic debris at global scale. Based on the spatial coverage of our data grid, here we provided a first-order estimate of the range of the surface plastic load in the Mediterranean, using a wide confidence interval to address small scale of spatial heterogeneity. Thus, calculating a high-range concentration from the 90th percentile and a low-range from average of concentrations uncorrected for mixing by wind, the Mediterranean surface plastic load ranges from 756 to 2,969 tons.

Plastic accumulation in the Mediterranean Sea likely results from significant plastic input combined with a limited export to the Atlantic Ocean. At global scale, the Mediterranean Sea acts as a convective basin. The surface inflow of light Atlantic water is transformed into denser, deeper outflow of Mediterranean water due to evaporation greatly exceeding precipitation and river runoff [24]. The net water flow through the Strait of Gibraltar in the upper surface layer (10 m depth) at annual scale is estimated to be in the order of 10^5^ m^3^ s^-1^ toward the Mediterranean Sea [27]. This hydrodynamic pattern suggests that a proportion of the floating plastic pollution in the Mediterranean may originate outside the basin, with the Mediterranean Sea acting as a sink for Atlantic floating plastic pollution. This proposition can be tested with input-output measurements of plastic flow across the Strait and accounting for the small time scale of variability associated with wind and wave action in this area.

The comparatively high abundance of plastic items larger than 20 mm found in the Mediterranean Sea (Fig. 1) could be explained by the shorter pathways that large plastic objects need to travel in the basin, compared to the longer distances needed to reach accumulation zones in subtropical ocean gyres. There are no estimates of plastic input for the Mediterranean Sea. Recently, the plastic input into the Black Sea via the Danube River was conservatively estimated at 1,533 tons per year [28]. Given that debris released by the Danube should be on the order of the Nile River alone [8], and that plastic also enters into the Mediterranean from other numerous terrestrial and maritime sources, the load of floating plastic accumulated into the Mediterranean basin seems relatively low in relation to the expected inputs.

Several studies have reported an abundance of large buoyant plastic objects on the Mediterranean seafloor [29, 30]. Large plastic objects (e.g., bags, and bottles) often show large cavities that facilitate their ballasting with suspended sediments and their colonization by organisms that use these floating objects as substrates or refuges. The diversion of large plastic objects to the seafloor likely contributes significantly to reduce the plastic load at the surface. Nevertheless, the size distribution of plastic items in the Mediterranean Sea also suggests important removal of microplastics. The paucity of small plastic particles (< 2 mm) in spite of the abundance of large objects (> 20 mm) suggests a particularly high removal rate of microplastic in the Mediterranean surface waters. Removal mechanisms of microplastics include ingestion by planktivorous animals and ballasting by biofouling [5, 31, 32], and these could be greater in the Mediterranean, where ecosystem production is higher than in the subtropical gyres. However, estimates of ingestion rates of microplastic by marine life or microplastic abundance on the seafloor are still needed to test this hypothesis.

The historical time series of plastic concentration in surface waters of other world regions initially kept pace with increasing plastics production, but plastic concentrations seem to have leveled off over the last three decades [1, 4, 31]. Whereas there are no long time series for plastic debris measured in the Mediterranean, combining available visual censuses of floating debris, Suaria and Aliani recently reported no clear temporal trend in surface plastic concentration since 1980 [19]. However, it is striking that the highest visual count of surface plastic debris in the Mediterranean was reported in the earliest assessment, conducted in 1979 in the center of the basin, near Malta [15]. In this visual assessment, Morris reported around 1,300 plastic items km^-2^, at least one order of magnitude higher than all other visual counts carried out between 1986 and 2013 [16–19]. This result could be due to the fact that the counting protocol used by Morris, with small observation area and short survey times during an exceptionally calm period, was particularly suitable to detect small debris. He calibrated the minimum size detectable by the observer to be 1.5 cm. We measured 13,614 items larger than 1.5 cm per km^2^ (range: 4,241–31,811 items km^-2^) in the central Mediterranean region, suggesting an order-of-magnitude increase in the surface plastic concentration between 1979 and 2013, comparable to the increase estimated in the North Pacific accumulation zone between the 1972–1985 and 2002–2012 sampling periods [4]. An initial rise in plastic pollution in the Mediterranean followed by a subsequent period of steady concentrations would agree with the patterns found in other ocean regions [1, 4, 31], but the limited spatial and temporal resolution of the data and the different assessment methods (visual counts vs. net tows) precludes a robust inference on the temporal trend of plastic pollution. The size distribution of floating plastic debris shows a power increase in plastic abundance from large objects to millimeter-sized particles. Therefore, visual counts strongly depend on minimum plastic size detectable by the observer, which is related to sea state, ship speed, observation distance and observer concentration [16, 19, 33].

The Mediterranean Sea represents less than 1% of the global ocean area, but has disproportionate ecological and economic values. It harbors between 4% and 18% of all marine species [34]; and fishing industry, aquaculture, maritime transport and coastal tourism are key sources of income for the Mediterranean nations [13]. For this reason, the possible impacts of the plastic pollution may be particularly relevant in the Mediterranean. We know that plastic pollution may affect marine ecosystems and economic activities in different modes [35–38], although little is yet known about the magnitude of the implications of this pollution. Among the possible impacts, those related to the accidental ingestion of plastic debris by marine life are of particular concern since they may involve a wide array of marine taxa. In the Mediterranean Sea, plastic debris has been found in stomachs of small fish [20], seabirds [39], turtles [40] and sperm whales [41]. Recently, nanoplastic particles (in the order of tens of microns in size) were found in significant amounts in oysters and mussels cultured on the coasts of northern Europe [42]. In addition to gastrointestinal blockages and other harm derived from plastic debris ingestion, even including mortality [39–41], ingested plastics may contain high levels of toxic compounds added during manufacture or absorbed from seawater. Plastic debris absorbs contaminants, including bioaccumulative compounds, about one hundred times more efficiently than naturally occurring suspended organic matter [36]. Several studies suggest that some plastic-associated contaminants may be transferred to organisms during digestion [36, 43], and recent laboratory experiments indicate that plastic-associated contaminants may alter endocrine system function of fish [44]. In the ocean, high concentrations of plastic-associated contaminants (e.g., phthalates, and nonylphenol) have been measured in small planktivorous fish of the North Pacific Subtropical Gyre [45] or in large filter-feeding organisms (basking shark and fin whale) of the Mediterranean Sea [46]. There are signs enough to suggest that chronic exposure of planktivorous animals to microplastic pollution could have extensive toxicological impacts on organisms living in the plastic accumulation regions, a threat requiring special attention in the rich Mediterranean ecosystem.

## Conclusions and Final Remarks

In the present work, we identify the Mediterranean Sea as a great accumulation zone of plastic debris. From the averaged plastic concentration measured into the basin, the surface load of plastic in the Mediterranean is estimated to be around one thousand tons, increasing the estimated global load of surface plastic by 7% [5], in agreement with the relative loads predicted for this Sea by modeling at the global scale [8]. However the calibration of distribution models to upscale absolute large-scale loads of plastic must be treated with caution due to the low agreement found between measurements and model predictions within the basin scale (Fig. 2). The estimate of plastic load in the Mediterranean Sea derived from model calibration [6] was one order of magnitude higher than our estimate from a gridded approach, considering both total and microplastic (< 5 mm) loads. The development of more accurate estimates of the magnitude and sub-basin distribution of the plastic pollution in the Mediterranean Sea requires improved sampling resolution and coverage.

Model simulations are shown here to be useful tools to guide field surveys aimed at assessing the magnitude of global marine plastic pollution. The model by Lebreton et al. [8] identified the Mediterranean Sea as a region of high load of plastic pollution, and this is confirmed by our estimates. Lebreton and coworkers also pointed out the Bay of Bengal and the South China Sea as relevant accumulation zones, while the model of van Sebille and coworkers [9] drew attention to the Barents Sea in the Arctic Ocean as an accumulation zone. Recent measurements verified the existence of high plastic abundance in areas of the Bay of Bengal [33], but current assessments of plastic concentrations in the South China Sea [47] suggest lower loads than predicted. Available data for floating debris in Arctic waters show relatively low plastic concentrations [5, 10], but few measurements are yet available and these assessments need be extended to higher latitudes. Interestingly, recent analyses of plastic pollution in ice cores show significant loads of microplastics in the Arctic ice sheet [48], which implies accumulation of plastic pollution in the Arctic Ocean.

The discovery of large-scale accumulations of marine debris has attracted worldwide attention in the media, which often refer to these areas as “great garbage patches”. However, these marine plastic accumulations are inaccurately illustrated in some media reports. The present work converges with other studies [1, 4–9] to define these accumulation zones as very large spans of the ocean (millions of km^2^ in area), although their borders are diffuse and changing, and their interior shows high heterogeneity at multiples scales [8, 26]. These accumulation zones are dominated by tiny plastic pieces, mainly on the order of millimeters, not easily perceptible by an observer on a ship. When the sea is calm, plastic fragments are present in nearly 100% of net surface tows in these areas, each covering around 1000 m^2^, but the density of plastic pieces is not as high as the term “patch” may suggest. The typical mean spatial concentration measured with net tows is around 1 plastic item in 4 m^2^, reaching 1–10 items m^-2^ in the most polluted areas.

Marine plastic pollution has spread to become a problem of planetary scale after only half a century of widespread use of plastic materials, calling for urgent management strategies to address this problem. Cleanup activities on the shoreline could be particularly effective in the Mediterranean Sea since shore deposition of floating debris must be common in this semi-closed sea [49]. However, as the production of plastic materials will likely continue to increase in the coming decades [50], management strategies should be addressed at the pollution sources in order to prevent the release of plastic discards to the environment.

## Supporting Information

S1 Fig Photograph of typical textile fiber (red) and fishing thread (blue). (TIF)

S2 Fig Relationship between mass (*M*) and numerical (*N*) concentrations of floating plastic debris. Red circles correspond to surface tows carried out in the Mediterranean Sea (*n* = 39), and blue circles to the data set compiled by Cózar et al. for the global ocean [5] (*n* = 571). Black line shows the log-log linear-square fitting on all data in plot (log *M* (g km^-2^) = 1.22 log *N* (items km^-2^)- 4.04; *n* = 609, *r* = 0.8571, *p* < 0.0001).
(TIF)
