# Supplementary figures and images for: Plastic Accumulation in the Mediterranean Sea
Source: PLoS One. 2015 Apr 1;10(4):e0121762. doi: 10.1371/journal.pone.0121762 (PMC4382178; doi:10.1371/journal.pone.0121762)

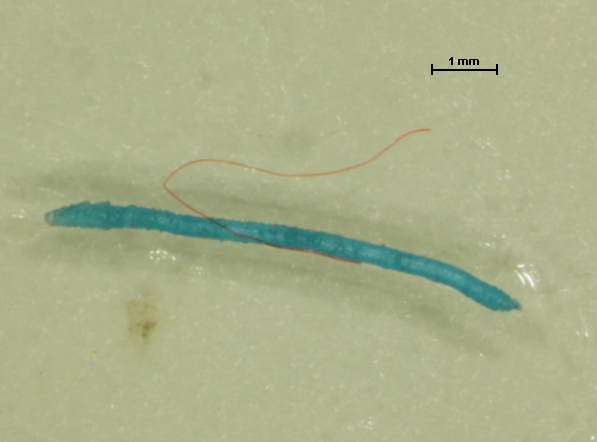

Supplement: S1 Fig — (TIF) [file pone.0121762.s001.tif]

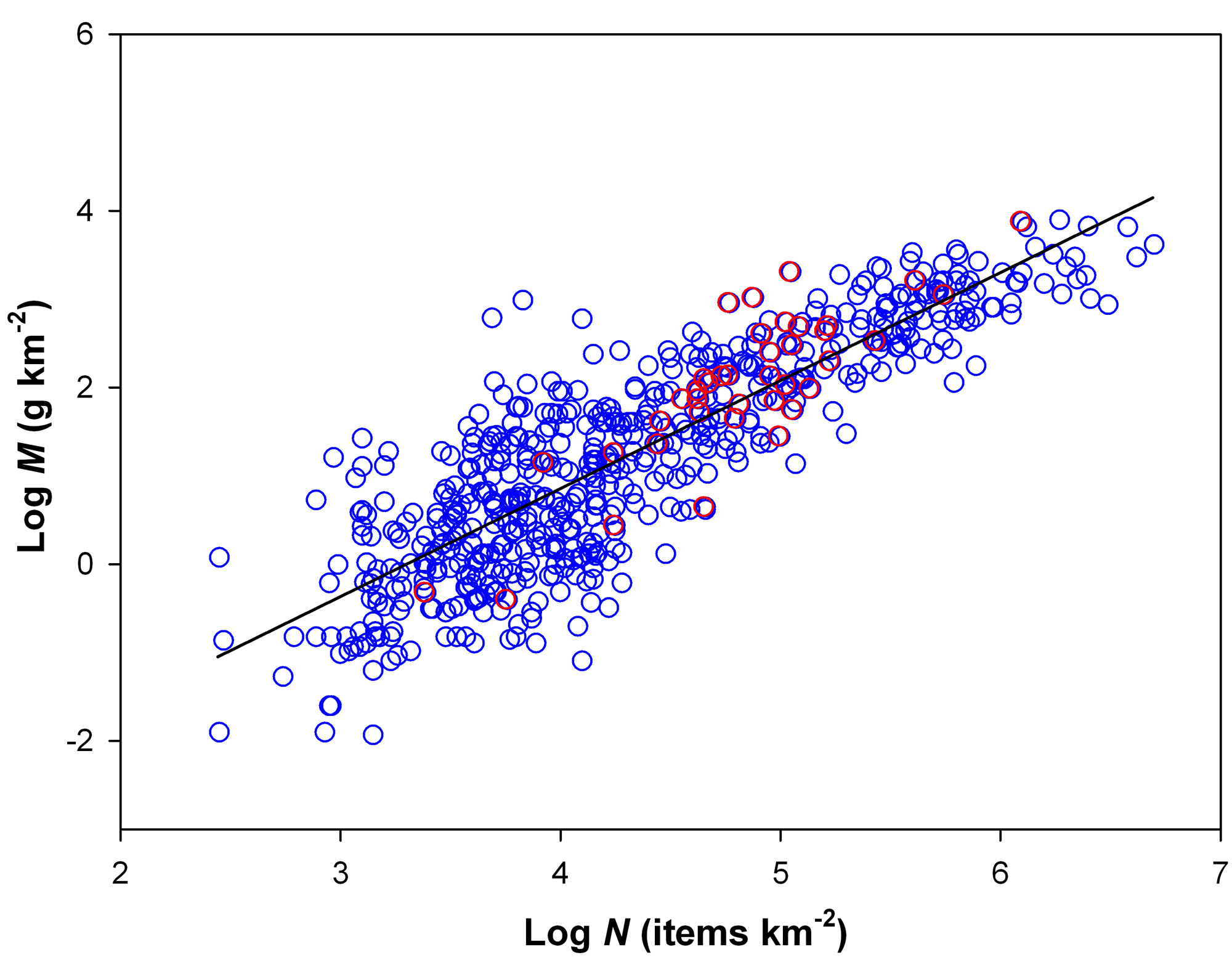

Supplement: S2 Fig — Red circles correspond to surface tows carried out in the Mediterranean Sea (n = 39), and blue circles to the data set compiled by Cózar et al. for the global ocean [5] (n = 571). Black line shows the log-log linear-square fitting on all data in plot (log M (g km-2) = 1.22 log N (items km-2)- 4.04; n = 609, r = 0.8571, p < 0.0001). (TIF) [file pone.0121762.s002.tif]
